# Effects of Feeding Multinutrient Blocks Including Avocado Pulp and Peels to Dairy Goats on Feed Intake and Milk Yield and Composition

**DOI:** 10.3390/ani10020194

**Published:** 2020-01-23

**Authors:** Trinidad de Evan, María Dolores Carro, Julia Eugenia Fernández Yepes, Ana Haro, Lesly Arbesú, Manuel Romero-Huelva, Eduarda Molina-Alcaide

**Affiliations:** 1 Departamento de Producción Agraria, Escuela Técnica Superior de Ingeniería Agronómica, Agroalimentaria y de Biosistemas, Universidad Politécnica de Madrid, Ciudad Universitaria, 28040 Madrid, Spain; t.deevan@alumnos.upm.es (T.d.E.); mariadolores.carro@upm.es (M.D.C.); 2 Estación Experimental del Zaidín (Consejo Superior de Investigaciones Cientificas), Profesor Albareda 1, 18008 Granada, Spain; julia.fernandez@eez.csic.es (J.E.F.Y.); ana.haro@eez.csic.es (A.H.); leslyarbesu@gmail.com (L.A.); 3 NUTROFAR, SL, Parque Empresarial Los Llanos, Calle Galicia, No 270. 41909 Salteras, Sevilla, Spain; manuel.huelva@hotmail.com

**Keywords:** avocado pulp, avocado peels, dairy goats, milk fatty acids

## Abstract

**Simple Summary:**

The high and volatile prices of conventional ingredients for animal feeding has directed the attention of ruminant nutritionists toward local alternative resources such as agroindustrial by-products and fruits wastes. The inclusion of these resources in the diet might contribute to reducing feeding costs and environmental issues associated with both livestock production and by-products and wastes accumulation might be prevented. The global production and consumption of avocado have risen sharply in recent years, partly due to the recognition of its health-promoting potential in humans. The increased consumption of avocado and its derivatives is producing great amounts of wastes and by-products that might be reutilized in ruminant feeding. Our hypothesis was that avocado wastes (a mixture of pulp and peels) could be included in multinutrient blocks for dairy goats and improve the quality of the milk fatty acid profile without negatively affecting milk yield. However, the intake of multinutrient blocks containing 14.8% avocado wastes was low probably due to avocado lipids oxidation and rancidity. No changes were observed in milk production, but feeding blocks with avocado wastes increased milk fat content with only subtle changes in the fatty acid profile of fat milk.

**Abstract:**

Twelve Murciano-Granadina dairy goats were divided into two homogeneous groups, which were fed either a control diet composed of 40% alfalfa hay and 60% concentrate or a diet based on 40% alfalfa hay, 40% concentrate and 20% multinutrient blocks, including 14.8% avocado pulp and peels (APP). Total dry matter (DM) intake was similar (*p* = 0.709) for both diets, but APP-fed goats had lower (*p* = 0.024) concentrate intake and tended (*p* = 0.063) to have lower fat intake compared with those fed the control diet. The average intake of blocks was low (66.4 g DM/d), which was attributed to avocado lipids oxidation and rancidity. Neither milk yield (*p* = 0,921) nor the efficiency of energy and nitrogen use were affected (*p* = 0.909 and 0. 840, respectively) by the diet, but milk fat tended to be greater (*p* = 0.057) in the APP-fed goats compared with the animals fed the control diet. Other milk components were similar (*p* ≥ 0.110) for both diets, and only subtle changes in the milk fatty acid profile were observed. In summary, the intake of blocks containing avocado wastes by dairy goats was low probably due to avocado lipids oxidation causing off-flavors and reduced palatability.

## 1. Introduction

Dairy goat production is of increasing interest in the Mediterranean basin due to the exponential growth of market demand for goat milk and derived products [1]. The increased demand has promoted more intensive production systems, in which animal feeding is frequently based on imported feed ingredients. The high and volatile prices of feed ingredients in the recent past has caused producers to search for alternative feed sources, such as agro-industrial by-products and fruits wastes, in order to reduce production costs without decreasing milk yield and quality [2,3]. Additionally, environmental issues associated with both livestock production and by-products or fruits wastes accumulation could be alleviated by using them in animal feeding [4].

The global production and consumption of avocado (*Persea Americana*; Hass variety) has risen sharply in recent years, partly due to the recognition of its health-promoting potential in humans [5] About 6 × 10^6^ tonnes of avocado are produced worldwide per year, with Mexico being the greatest producer [6] but it is estimated that about 24% of the original fruits becomes waste [7]. The waste and by-products of avocado usually have a high-moisture content, are fibrous, and may contain fermentable materials, all which result in rapid spoilage under aerobic conditions. Additionally, the disposal of these wasted and by-products is concentrated in the harvest season (6–8 months per year). Seasonal limitations and the high-moisture content, which complicates preservation, are therefore the main obstacles for using avocado wastes and by-products as common ingredients in animal feeding, despite the fact that avocado may contain several bio-active compounds such as essential oils and phenols [8] which may have health benefits for the animals.

Hernández-López et al. [9] observed that including avocado wastes in a mixed diet for finishing pigs reduced the lipid content in the muscle Longissimus thoracis et lomborum, increased the degree of fatty acid (FA) unsaturation and reduced both lipid and protein oxidation rates during chilled meat storage. In broiler chickens, van Ryssen et al. [10] observed that the inclusion of 7.3% to 29.3% dried avocado meal in the diet reduced the feed intake and the growth of the animals. Whereas some data are available on the composition, rumen in situ degradability and digestibility of avocado meal and pulp for ruminants [11,12], to our best knowledge, no information exists on the effect of feeding avocado wastes to dairy ruminants. Our hypothesis was that a mixture of avocado pulp and peels could partially replace conventional ingredients in a concentrate for dairy goats, decreasing feeding costs and improving milk yield and/or the FA profile of milk. The aim of the present work was therefore to study the effect of replacing conventional feed ingredients in the concentrate of dairy goats with a mixture of avocado pulp and peels (APP) on feed intake and milk yield and composition.

## 2. Materials and Methods

The goats used in this study were cared for and handled in accordance with the Spanish guidelines for experimental animal protection [13] in line with the European regulations. All the experimental procedures were approved by the Ethic Committee for Animal Experimentation of the Spanish Research Council and the Junta de Andalucía (Approvals Numbers 24/05/2016/091 and 22/06/2016/115, respectively).

### 2.1. Animals and Diets

Twelve Murciano-Granadina dairy goats in the middle of the first lactation were selected and divided into 2 homogeneous groups of 6 goats each based on body weight (48.4 ± 2.40 kg), average voluntary feed intake (66.7 g dry matter (DM)/kg BW^0.75^), and milk yield (790 g milk/d) at the beginning of the experiment. Each group was randomly assigned to one of the experimental treatments: a control diet composed of 40% alfalfa hay and 60% concentrate, and a diet composed of 40% alfalfa hay, 40% concentrate and 20% multinutrient blocks including a mixture of avocado pulp and peels (APP). The ingredient composition of the concentrate and the multinutrient blocks is shown in Table 1.

Waste fruits of avocado were processed by separating the pulp, peels and seeds and the weight of each fraction was recorded. The APP mixture contained 81.3% and 18.7% pulp and peels, respectively, representing their relative percentages in the fruit. The manufacturing of the multinutrient blocks followed the protocol of Ben Salem and Nefzaoui [14] with the modifications described by Molina-Alcaide et al. [15]. Briefly, all the ingredients were mixed, water was added and the mixture was packed into metal molds and hard-pressed. Blocks were then removed from the molds, air-dried outdoors (5 to 7 days), and stored at room temperature until feeding.

The specific nitrogen (N) and energy requirements of Murciano-Granadina goat breed [16] were considered in the diets formulation. During the adaptation period, diets were fed at 84 g DM per kg BW^0.75^ and refusals represented between 20% and 25% of the distributed feeds. During the experimental period, the animals were fed at the same rate.

### 2.2. Experimental Procedures

Animals were hosed in floor individual boxes and had free access to fresh water over the trial. Goats were fed once daily (09:00 h) the corresponding diet and were milked once a day in the morning in a 1 × 10 stall milking parlour (DeLaval, Madrid, Spain). After 25 days of diet adaptation, the individual intake of each feed and milk production were registered during a 7-day sampling period. Refusals of alfalfa hay, concentrate and multinutrient blocks from each animal were collected and weighed daily, pooled by animal, and stored at −20 °C until chemical composition analyses. Feed intake was calculated as the difference between the amount of each feed supplied and the corresponding refusals. During the sampling period, milk density was daily measured and aliquots were stored at −30 °C (without preservatives) for analysis of chemical composition and FA profile.

### 2.3. Chemical Analyses

Dry matter (method 934.01), ash (method 942.05), ether extract (EE; method 920.39), contents in samples of supplied feeds and refusals were analyzed according to Association of Official Analytical Chemists [17] while N was analyzed by total combustion according to the method LECO^®^/Dumas. The analyses of neutral detergent fiber (NDF), and acid detergent fiber (ADF) were carried out according to Van Soest et al. [18]. using an Ankom220 Fiber Analyzer unit (Ankom Technology Corp., Macedon, NY, USA). For the NDF analysis of concentrate and multinutrient blocks samples, α-amylase was used. Lignin was determined by solubilization of cellulose with 72% sulfuric acid [18]. All the results were expressed exclusive of residual ash. The gross energy content of the feeds offered and the refusals was determined in an adiabatic calorimeter (model 1356; Parr Instruments Co., Moline, IL, USA).

Additionally, free, protein-bound, and fiber-bound condensed tannins in the APP mixture were sequentially extracted according to the procedure of Perez Maldonado and Norton [19] and condensed tannins from quebracho powder (Roy Wilson Dickson Ltd., Mold, UK) were used as the standard for quantification. The content in total extractable polyphenols of the APP mixture was analyzed by the Folin-Ciocalteu assay as described by Singleton et al. [20].

Milk total solids content was determined by freeze-drying of milk samples. The total N content in milk was determined by the Dumas method using TruSpec CN equipment (Leco Corp. St. Joseph, MI, USA), whereas the content in non-protein N and non-casein N was analyzed in the filtrate obtained after precipitation of milk samples with trichloroacetic acid (12%, weight/volume) and acetic acid (10%; weight/volume) at pH 4.1, respectively [21].

The fat content of milk was measured by the Gerber method [22]. Extraction of total FA in feed samples was performed according to the procedure of Folch et al. [23] and the FA were methylated according to Kramer and Zhou [24] with slight modifications, as double methylation was carried out, using first NaOH/methanol, at 50 °C for 15 min, followed by HCl/methanol at 50 °C for 1 h to obtain the FA methyl esters (FAME). The procedures for extraction and transesterification of milk FA have been detailed by Abecia et al. [25]. The FAME were separated and quantified as described by Shingfield et al. [26] using a Focus gas chromatograph (Thermo Scientific, Milan, Italy) provided with a flame-ionization detector, a 100-m fused silica capillary column (0.25 mm i.d., 0.2-μm film thickness; SP-Supelco, Bellefonte, PA, USA) and helium as carrier gas.

### 2.4. Calculations and Statistical Analyses

The N content of feeds and refusals was converted to crude protein (CP) multiplying by 6.25, whereas CP in milk was calculated by multiplying the N content by 6.38. The content of milk in protein-N was calculated as the difference between total N and non-protein N, and the content in casein-N was estimated as the difference between total N and non-casein N. The whey-N content in milk was calculated as the difference between milk protein content and casein-N. Finally, the milk lactose content was estimated as the difference between total solids content and the content in protein, fat and ash.

Data were analysed by one-way variance analysis using the PROC GLM of SAS [27]. Differences were considered significant at *p* < 0.05, and *p* values between 0.05 and 0.10 were declared as trends and discussed.

## 3. Results and Discussion

The APP mixture used in our study was composed of 81.3% pulp and 18.7% peels, which agrees well with the proportion of 80.2% and 19.8% of pulp and peels in the Hass avocado, respectively, reported previously [28]. Avocado fruits are rich in bioactive compounds, and it has been shown that their content varied with both the avocado fraction (peels, pulp and seeds) and cultivar variety [28]. In our study, the content of the APP mixture in free, protein-bound, and fiber-bound condensed tannins was 5.90, 1.76 and 15.1 g/kg DM, respectively, and the content in total condensed tannins (22.8 g/kg DM) was only slightly greater than that of total extractable polyphenols (18.5 g/kg DM). Wang et al. [28] analyzed the polyphenols content of the different fractions of eight avocado cultivars, and observed that pulp had lower content (4.0 to 32.7 g/kg DM) compared with the peels (19.5 to 57.3 g/kg DM) and seeds (98.4 to 161 g/kg DM). For all fractions analyzed, Hass variety had greater polyphenols content than the other varieties. A similar polyphenols content in Hass avocado peels (63.5 g/kg DM) has been reported by Tremocoldi et al. [29] and in Fuerte avocado pulp (4.0 g/kg DM) by Daito et al. [30]. The polyphenols content observed in our study for the APP mixture is consistent with the values reported by these authors for each individual fraction.

The chemical composition and FA profile of the APP mixture, alfalfa hay, concentrate and multinutrient blocks are shown in Table 2. The chemical composition of the APP mixture agrees well with the values reported by others [31,32] for avocado pulp, which is characterized by its high fat content, frequently ranging from 11% to 23% of the fresh matter [31,33]. In agreement with previous studies on avocado composition [5,32,34] oleic acid was the most abundant FA in the APP mixture, followed by linoleic, palmitic, vaccenic and palmitoleic acids. As a consequence, the monounsaturated FA (MUFA) represented about 2/3 of total FA, whereas saturated (SFA) and polyunsaturated (PUFA) FA were only 15.9% and 16.2% of total FA, respectively.

The concentrate and the multinutrient blocks were formulated to have similar CP and EE contents, but the blocks resulted in lower EE and greater NDF content compared with the concentrate (Table 2). The lower EE content of the blocks was attributed to fat losses during manufacturing, possibly as a consequence of the effluent and small particles losses produced during the hard-pressing of block ingredients. There were marked differences in the FA profile of the concentrate and the multinutrient blocks, whereas the PUFA were the most abundant FA in the concentrate (52.5% of total FA), the MUFA were the predominant FA in the multinutrient blocks (58.6% of total FA). The high proportion of PUFA in the concentrate can be explained by the inclusion of cotton and sunflower seeds, as their lipids represented about 53.5% of total EE in the concentrate and both corn and sunflower oils are highly unsaturated, with about a 2:1 ratio of PUFA to SFA [35,36]. The concentrate used in our study had a 2.4/1 ratio of PUFA to SFA, and this high value can be explained by the high proportion of corn (360 g/kg), as corn contributed to 24.1% of total EE in the concentrate and its oil has high PUFA/SFA ratio (over 4.5/1; [37]). The lipids supplied by the APP mixture manufacturing, and the high content in MUFA of the blocks is consistent with the FA profile of the APP mixture.

As shown in Table 3, there were no differences (*p* = 0.723) between groups in the intake of alfalfa hay, but the partial replacement of concentrate by the multinutrient block reduced the daily intake of concentrate (*p* = 0.024); however, the intake of multinutrient block was lower than the reduction observed in the concentrate, as daily blocks intake averaged 66.7 g per goat. To our best knowledge, there is no information on the avocado palatability for goats, but it has been reported that the inclusion of 7.3% to 29.3% of dried avocado meal in the diet reduced the feed intake and the growth of poultry [10]. In contrast, no reductions in either feed intake or animal performance were observed by including 30% of a paste made of ground avocado fruit wastes in a mixed diet for finishing pigs [9]. These results might indicate that avocado palatability, or even tolerance, varies with the animal species. In fact, many varieties of avocado contain persin, an oil-soluble compound that is toxic to several animal species but especially to birds [38,39]. Persin is concentrated in the seeds of avocado [40] and both the fact that no avocado seeds were included in the multinutrient blocks and the low level of inclusion of the APP mixture seems to preclude the hypothesis that avocado toxicity was responsible for the low intake of the multinutrient blocks.

Eliyahu et al. [12] observed that avocado pulp stored outdoors (25 °C of average temperature) in containers covered with paper sheets was rapidly contaminated by mold and yeast within the first 3 days of storage despite of its low initial pH (4.52). These authors observed that the pH of avocado pulp was increased from 4.52 to 8.30 over the first 7 days of storage, whereas the in vitro DM digestibility the water soluble carbohydrates content was reduced from 30% to 22% and from 15.3 to 1.62 g/kg DM, respectively. The multinutrient blocks used in the present study were dried outdoors and their low moisture content (926 g DM/kg) prevented the growth of mold and yeast, but the oxidation of avocado lipids probably caused rancidity and reduced the palability of the blocks. In fact, avocado pulp is highly sensitive to post-harvest oxidation, which results in rancidity and subsequent production of undesirable off-flavors [41]. It has been reported that the high chlorophyll content of avocado lipids can act as a proxidant by stimulating photo-oxidation [42]. In contrast, to our results, Eliyahu et al. [12] reported that Assaf lambs weighting 50 ± 1.5 kg consumed daily 1.0 kg DM of a total mixed diet containing 49.6% fresh avocado pulp. The high avocado intake observed in their study would indicate a high palatability of fresh avocado that has not underwent lipid oxidation, as may have happened in our study. Despite the low intake of blocks in the APP-fed goats, there were no differences between the groups (*p* = 0.132 to 0.867) in the intake of any measured nutrient with the exception of EE which was lower (*p* = 0.005) for the APP group than for the goats fed the control diet (Table 3).

As shown in Table 4, no differences between groups were detected in their initial body weight (*p* = 0.154), but APP-fed goats had la ower (*p* = 0.012) body weight than control ones by the end of the trial, indicating a mobilization of reserves. No differences were observed in milk yield and composition, excepting milk fat content and gross energy that tended (*p* ≤ 0.057) to be greater in the goats fed the APP diet compared with those fed the control diet. The mobilization of reserves observed in the APP-fed group is consistent with the lower concentrate intake but similar milk yield in the AAP-fed group compared with the control goats. However, results on milk yield and composition should be interpreted with caution due to the low number of goats in each experimental group, as differences between the two experimental groups might also be due to the random variation between goats. For both groups, milk composition was in the range of values previously reported for Murciano-Granadina goats in the middle of lactation [3,43]). Values of energy and N use were also similar to those reported previously for dairy goats in mid lactation [3,43] and were not affected by the diet (*p* ≥ 0.348). The low efficiency of N use indicates that the CP content in the diets used in our study was in excess of goat requirements.

The partial replacement of concentrate by the APP blocks caused only small changes in milk FA profile (Table 5). The milk from the APP-fed goats had a greater (*p* ≤ 0.050) proportions of caprylic (C8:0), heptadecanoic (C17:0 *iso*), elaidic (C18:1 *trans*-9) and eicosadienoic (C20:2 *cis* 11,14) acids, and tended (*p* ≤ 0.097) to greater proportions of caproic (C6:0) and docosanoic (C22:0) compared with the milk from control goats, but there were no differences between groups in the proportion of any other FA. The lack of differences (*p* ≥ 0.451) between diets in the content in <16C, 16C and >16C FA in the milk indicates similar FA uptake by the mammary gland in both groups of goats. Finally, neither the Σn6/ Σn3 ratio nor the atherogenicity index (calculated as described by Ulbricht and Southgate [44]) were influenced (*p* ≥ 0.585) by the diet. Altogether, these results indicate that feeding the APP blocks did not change milk fat quality. Differences in milk FA profile can be due to differences in FA intake, but also to other factors such as energy intake and ruminal conditions that influence unsaturated FA biohydrogenation [45]. The similar energy and FA intake in both groups in our study, caused by the low intake of the APP blocks, would justify the negligible differences between groups observed in the FA profile.

## 4. Conclusions

The mixture of avocado peels and pulp is a high-moisture by-product, but its DM is rich in high-oleic fat, low in fiber and has a medium protein content. Intake of this mixture, included in multinutrient blocks at 14.8%, was low, which was attributed to avocado lipids oxidation causing off-flavors and reduced palatability. The mixture of avocado pulp and peels did not change either milk yield or the efficiency of energy and N use, but tended to increase milk fat content and caused only subtle changes in milk fatty acid profile. It would be convenient to formulate strategies that attenuate the oxidation of avocado lipids and thus avoid their possible negative consequences on the palatability of feed, which could condition their consumption.

## Figures and Tables

**Table 1:** Ingredient composition (g/kg fresh matter) of the concentrate and the multinutrient block including a mixture of avocado pulp and peels (APP) fed to dairy goats.

Ingredient	Concentrate	APP Multinutrient Block
APP mixture ^1^	-	148
Corn	360	220
Oat	125	-
Barley straw	-	80
Sunflower meal	65	250
Wheat bran	-	221
Sunflower seeds	30	-
Cotton seeds	80	-
Soybean meal	175	-
Molasses-glycerol	20	-
Sugar beet molasses	-	51.6
Sugar beet pulp	140	-
Calcium carbonate	-	20.0
Palm soap	-	0.4
Urea	-	4.0
Vitamin-mineral mixture	5.0	5.0

^1^ Mixed in the same proportion as in the original fruits (81.3% and 18.7% pulp and peels, respectively).

**Table 2:** Chemical composition (g/kg dry matter unless otherwise indicated) and fatty acid (FA) profile of the ingredients of the experimental diets.

Item	Avocado Pulp and Peels	Alfalfa Hay	Concentrate	Multinutrient Blocks
Dry matter, g/kg fresh	247	876	875	926
Organic matter	823	838	895	847
Crude protein	71.9	158	180	173
Neutral detergent fibre	90.1	477	269	410
Acid detergent fibre	62.9	339	118	244
Acid detergent lignin	4.0	89.8	23.8	95.7
Ether extract	640	25.2	49.9	38.7
Non-structural carbohydrates ^1^	21.0	199	345	239
Gross energy, MJ/kg dry matter	30.9	16.1	18.0	16.1
Fatty acid, g/100 g of identified fatty acids				
C10:0	0.785	0.598	0.476	0.006
C12:0	0.006	0.168	0.009	0.010
C13:0	0.013	0.058	0.027	0.022
C14:0	0.062	0.868	0.343	0.233
iso C15:0	0.002	0.016	0.001	ND
anteiso C15:0	0.019	0.257	0.068	0.280
C15:0	0.007	0.553	0.036	0.091
iso C16:0	0.027	0.015	0.021	0.258
C16:0	13.9	30.8	16.8	23.0
iso C17:0	0.144	0.017	0.0001	0.249
C17:0	0.002	0.572	0.091	0.141
anteiso C18:0	0.001	ND	0.002	0.099
C18:0	0.387	7.31	2.69	2.42
C20:0	0.050	1.34	0.261	0.344
C21:0	0.042	0.756	0.063	0.251
C22:0	0.393	1.74	0.310	0.435
C23:0	0.016	0.767	0.050	0.061
C24:0	0.057	3.83	0.160	0.391
C26:0	0.001	0.068	0.132	ND
Total saturated FA (SFA)	15.9	49.7	21.5	28.3
C14:1 cis-9 n5	0.001	0.048	0.002	D^2^
C16:1 cis-9 n7	4.90	0.440	0.317	3.15
C17:1 cis-10 n7	0.092	0.107	0.047	0.047
C18:1 trans-9 n9	0.010	0.317	0.145	0.401
C18:1 trans-11 n7	0.009	0.008	0.005	ND
C18:1 cis-9 n9	57.0	6.33	24.51	50.0
C18:1 cis-11 n7	5.83	0.334	0.863	4.47
C20:1 cis-11 n9	0.007	0.188	0.011	0.007
C22:1 cis-13 n9	0.007	4.74	0.051	0.398
C24:1 cis-15 n9	0.001	0.262	0.023	0.135
Total monounsaturated FA (MUFA)	67.9	12.8	26.0	58.6
C18:2 trans-9, trans-12 n6	ND ^2^	0.012	0.002	0.012
C18:2 cis-9, cis-12 n6	14.4	18.8	50.7	8.82
C18:3 cis-6,9,12 (ϒ) n3	0.201	0.171	0.235	0.389
C18:3 cis-9,12,15 (α) n3	1.49	16.7	1.17	0.344
C20:2 cis-11,14 n6	0.016	0.304	0.044	0.890
C20:3 cis-8,11,14 n6	0.015	0.696	0.072	1.376
C20:3 cis -11,14,17 n3	ND	0.076	0.023	0.000
C20:4 cis-5,8,11,14 n6	ND	0.000	0.001	0.255
C20:5 cis-5,8,11,14,17 n3	0.023	0.370	0.088	0.259
C22:2 n6	0.004	0.131	0.045	0.672
C22:5 cis-7,10,13,16,19 n3	ND	0.061	0.068	0.045
C22:6 cis-4,7,10,13,16,19 n3	0.003	0.177	0.011	0.062
Total polyunsaturated FA (PUFA)	16.2	37.5	52.5	13.1

^1^ Calculated as 1000—(neutral detergent fibre + crude protein + ether extract + ash); ^2^ ND: not detected.

**Table 3:** Average values of individual daily intake of feeds and nutrients in dairy goats fed the experimental diets.

Items	Diet ^1^		SEM ^2^	*p*-Value
	Control	APP		
Feeds (g dry matter)				
Alfalfa hay	536	573	30.9	0.506
Concentrate	748	541	21.1	0.024
Avocado multinutrient block	0	66.4	-	-
Total dry matter	1285	1180	48.3	0.709
Nutrients (g)				
Organic matter	1118	1021	37.1	0.132
Fat	50.9	43.6	1.52	0.005
Crude protein	219	199	8.07	0.165
Neutral detergent fibre	458	446	18.4	0.650
Acid detergent fibre	270	274	12.1	0.867
Acid detergent lignin	66.0	70.7	3.56	0.142
Gross energy, MJ/d	22.1	20.0	1.45	0.263

^1^ The control diet was composed of 40% alfalfa hay and 60% concentrate, and the APP diet was composed of 40% alfalfa hay, 40% concentrate and 20% multinutrient block including avocado pulp and peels; ^2^ SEM: Standard error of the mean.

**Table 4:** Average values of body weight and milk yield and composition in dairy goats fed the experimental diets.

Items	Diet ^1^		SEM ^2^	*p*-Value
	Control	APP		
Initial body weight, kg	49.6	47.6	0.976	0.159
Final body weight, kg	50.7	46.0	0.768	0.012
Milk yield, g/d	739	704	132.3	0.774
Milk composition, g/kg				
Fat	42.0	48.0	1.28	0.057
Protein	42.9	44.4	2.39	0.769
Casein	33.1	33.3	2.31	0.968
Whey protein	9.84	7.45	0.824	0.197
Lactose	58.1	48.4	2.63	0.115
Total solids	148	146	0.7	0.110
Gross energy, MJ/kg milk	3.53	4.07	0.125	0.055
Energy use efficiency (%) ^3^	12.0	13.4	1.89	0.348
Nitrogen use efficiency (%) ^3^	14.7	15.8	2.53	0.840

^1^ The control diet was composed of 40% alfalfa hay and 60% concentrate and the APP diet was composed of 40% alfalfa hay, 40% concentrate and 20% multinutrient block including avocado pulp and peels; ^2^ SEM: Standard error of the mean; ^3^ Calculated as either energy or nitrogen output in milk relative to energy or nitrogen intake, respectively.

**Table 5:** Fatty acid (FA) profile (g/100 g of identified FA) of milk fat from dairy goats fed the experimental diets.

Item	Diet ^1^		SEM ^2^	*p*-Value
	Control	APP		
C4:0	2.43	2.43	0.054	0.296
C6:0	2.94	2.96	0.0831	0.097
C8:0	3.70	3.86	0.090	0.021
C10:0	11.9	12.8	0.372	0.160
C11:0	0.293	0.297	0.016	0.139
C12:0	5.09	5.39	0.270	0.926
C13:0	0.195	0.201	0.016	0.396
C14:0	9.12	9.50	0.347	0.605
C15:0 *iso*	0.163	0.172	0.003	0.378
C15:0 ante *iso*	0.245	0.263	0.026	0.721
C15:0	0.783	0.809	0.034	0.443
C16:0 iso	0.191	0.172	0.010	0.567
C16:0	24.5	25.1	0.406	0.444
C17:0 *iso*	0.341	0.535	0.026	0.011
C17:0	0.634	0.613	0.018	0.606
C18:0 *anteiso*	0.196	0.169	0.023	0.928
C18:0	10.8	10.74	0.619	0.434
C20:0	0.176	0.177	0.009	0.419
C22:0	0.041	0.053	0.005	0.054
C23:0	0.023	0.017	0.0018	0.796
C24:0	0.015	0.014	0.001	0.196
C26:0	0.016	0.009	0.0012	0.620
Total saturated fatty acids	73.8	76.3	0.0002	0.619
C14:1 cis-9 n5	0.139	0.106	0.009	0.271
C16:1 trans-9 n7	0.011	0.021	0.003	0.267
C16:1 cis-9 n7	0.588	0.626	0.034	0.718
C17:1 cis-10 n7	0.007	0.002	0.002	0.460
C18:1 trans-9 n9	0.419	0.592	0.021	0.020
C18:1 trans-11 n7	0.429	0.563	0.0431	0.328
C18:1 cis-9 n9	19.5	17.1	1.11	0.498
C18:1 cis-11 n7	0.332	0.397	0.024	0.389
C20:1 cis-11 n9	0.080	0.095	0.012	0.678
C22:1 cis-13 n9	0.014	0.009	0.002	0.430
C24:1 cis-15 n9	0.017	0.012	0.002	0.620
Total monounsaturated fatty acids	21.5	19.5	1.13	0.576
C18:2 trans-9,12 n6	0.303	0.222	0.033	0.337
C18:2 cis-9,12 n6	2.28	2.86	0.167	0.179
C18:2 cis-9 trans-11 (CLA) n6	1.09	0.871	0.251	0.726
C20:2 cis-11,14 n6	0.0084	0.0297	0.0041	0.050
C22:2 cis-13,16 n6	0.046	0.011	0.025	0.568
C18:3 cis-6,9,12 n6	0.080	0.117	0.016	0.342
C18:3 cis-9,12,15 n3	0.196	0.249	0.031	0.493
C20:3 cis-8,11,14 n6	0.049	0.045	0.012	0.881
C20:3 cis-11,14,17 n3	0.017	0.009	0.0074	0.679
C20:4 cis-5,8,11,14 n6	0.225	0.204	0.018	0.632
C20:5 cis-5,8,11,14,17 n3	0.241	0.124	0.045	0.312
C22:4 cis-7,10,13,16 n6	0.0345	0.026	0.0030	0.316
C22:5 cis-7,10,13,16,19 n3	0.046	0.044	0.0035	0.808
C22:6 cis-4,7,10,13,16,19 n3	0.013	0.004	0.012	0.305
Total polyunsaturated fatty acids	4.43	4.82	0.332	0.856
According to the origin ^3^				
<16 carbon	37.0	39.4	1.26	0.451
16 carbon	25.8	25.0	0.576	0.522
>16 carbon	37.7	34.5	1.703	0.463
Σn3	0.513	0.43	0.060	0.585
Σn6	4.07	4.38	0.380	0.746
Σn6/Σn3	9.67	10.2	1.057	0.836
Atherogenicity index ^4^	2.69	2.66	0.209	0.949

^1^ The control diet was composed of 40% alfalfa hay and 60% concentrate and the APP diet was composed of 40% alfalfa hay, 40% concentrate and 20% multinutrient block, including avocado pulp and peels; ^2^ SEM: Standard error of the mean; ^3^ <16 carbon FA represent de novo synthesized FA; >16 carbon FA represent preformed FA taken up from circulation, and 16 carbon FA are derived from both sources. ^4^ Calculated as (C12:0 + (4 × C14:0) + C16:0)/unsaturated fatty acids according to Ulbricht and Southgate [44].
